# Spontaneous iliopsoas hematoma with hip pain and ecchymosis of the thigh

**DOI:** 10.1002/jgf2.279

**Published:** 2019-10-01

**Authors:** Fumihiko Takahashi

**Affiliations:** ^1^ Department of Cardiovascular Medicine Rumoi City Hospital Rumoi Japan

**Keywords:** computed tomography, ecchymosis, elderly, minor trauma, pain

## Abstract

We report an elderly patient with spontaneous iliopsoas hematoma. Primary care physicians should consider iliopsoas hematoma when patients complain of hip pain and thigh ecchymosis.

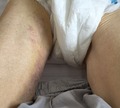

Hip pain is a common presentation in primary care. However, the differential diagnosis of hip pain is broad, presenting a diagnostic challenge.

An 87‐year‐old man was brought to the orthopedic clinic of our hospital accompanied by a nursing home staff. He was complaining of right hip pain for 5 days and ecchymosis of his right thigh. The patient had a medical history of diabetes and hypertension, which were managed with medical therapy for 5 years. He underwent bioprosthetic aortic valve replacement 4 years ago. He suffered from chronic, severe constipation for several months. Physical examination revealed tenderness of the right groin. Hip x‐ray revealed no fracture. Oral acetaminophen was prescribed for his hip pain. After 2 days, he was admitted to our hospital complaining of persistent hip pain and generalized malaise. On examination, ecchymosis was detected on his right groin and medial side of his right thigh (Figure 1). Laboratory tests revealed a hemoglobin level of 5.7 g/dL, a platelet count of 14.8 × 10^4^/µL and serum creatinine levels of 2.44 mg/dL. He was not on anticoagulant or antiplatelet therapy, and his coagulation profile was normal. Abdominal computed tomography (CT) revealed an approximately 5.4 × 5.8 × 20.0 cm right iliopsoas hematoma (Figure 2A). He was diagnosed with spontaneous iliopsoas hematoma because he denied any trauma to his hip. He was transfused with 4 units of red blood cells and was closely monitored. CT was repeated, and hematoma expansion was not observed. He was discharged on day 37, and his follow‐up abdominal CT scan on day 76 revealed reduced hematoma (Figure 2B).

**Figure 1:**
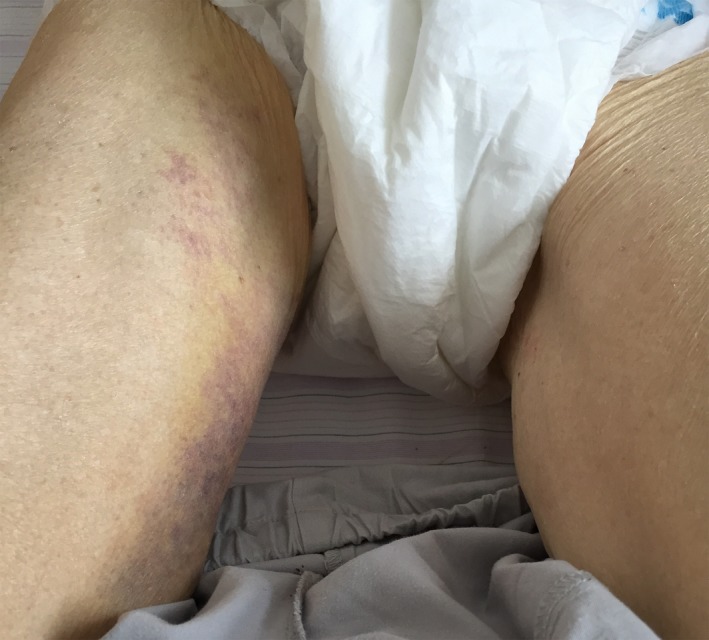
Ecchymosis on the inside area of his right thigh. The lesion varied in color from purple to yellow

**Figure 2:**
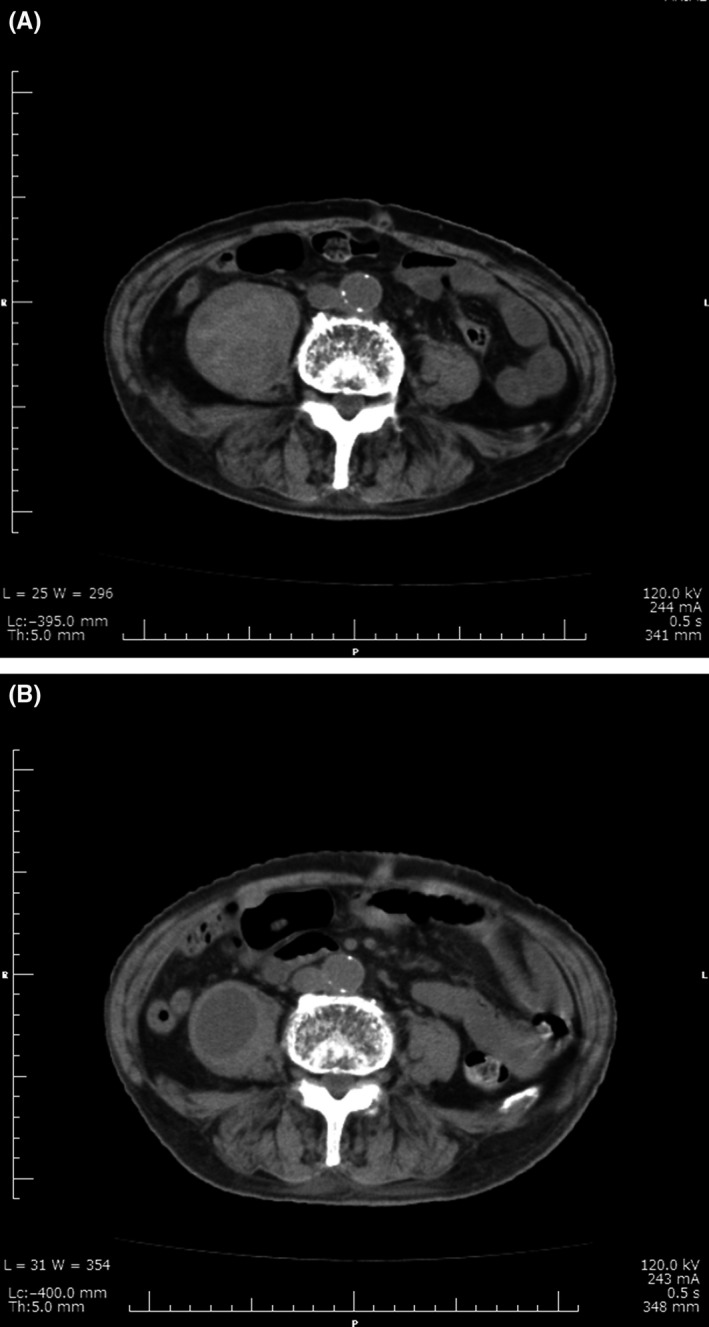
A, Computed tomography (CT) of abdomen and pelvis. CT revealed an approximately 5.4 × 5.8 × 20.0 cm right iliopsoas hematoma. B, Follow‐up CT of abdomen and pelvis. CT on day 76 revealed reduced hematoma

Iliopsoas hematoma is a potentially fatal and difficult‐to‐diagnose clinical entity. Spontaneous iliopsoas hematoma commonly occurs due to coagulopathy,^1^, ^2^ although the pathophysiology of spontaneous bleeding remains unclear. Moreover, the absence of coagulopathy does not rule out iliopsoas hematoma.^2^ Unrecognized minor trauma in vomiting or coughing suggested being an inciting factor in spontaneous retroperitoneal bleeding.^3^ Considering his chronic, severe constipation, repeated Valsalva maneuver during defecation might have been a minor trauma and have led to retroperitoneal bleeding. Besides, platelet dysfunction accompanied by his renal dysfunction could be a risk factor for bleeding.^4^ Ecchymosis of flank, groin, or thigh may be markers of potentially serious internal bleeding, although it is less common.^2^, ^5^ Therefore, primary care physicians should consider iliopsoas hematoma when patients complain of hip pain and ecchymosis of the thigh and groin. Early CT imaging is effective in diagnosing iliopsoas hematoma.

## CONFLICT OF INTEREST

The authors have stated explicitly that there are no conflicts of interest in connection with this article..
